# Canine polyostotic B-cell lymphoma: a case with clinical, immunohistochemical, and flow cytometric characterization, and review of the literature

**DOI:** 10.1177/10406387251329020

**Published:** 2025-03-21

**Authors:** Matthew Kornya, Connor Bryant, Brandon Lillie, Sebastien Sanz, Kristiina Ruotsalo, Dorothee Bienzle

**Affiliations:** Departments of Clinical Studies, Ontario Veterinary College; Pathobiology, Ontario Veterinary College; Pathobiology, Ontario Veterinary College; Departments of Clinical Studies, Ontario Veterinary College; Animal Health Laboratory, University of Guelph, Guelph, ON, Canada; Pathobiology, Ontario Veterinary College

**Keywords:** bone, cytology, dogs, histopathology, lymphocyte, lymphoma, monostotic, osseous

## Abstract

An 8-mo-old Mastiff-cross dog with bone pain and lytic-proliferative lesions in the radius, ulna, femur, vertebral spinous processes, and ribs, was diagnosed with lymphoma. The dog also had anemia and thrombocytopenia, and atypical circulating lymphocytes were identified as B cells by flow cytometry. Due to the multicentric, rapidly progressive disease, the dog was euthanized. Postmortem examination confirmed extensive bone replacement by lymphoma, and infiltration of lymph nodes, spleen, and liver. Histomorphology and immunohistochemistry showed a diffuse large B-cell lymphoma that was immunopositive for PAX5 and CD20, and immunonegative for CD3. Lymphoma of bone is rare in dogs and humans, and is most frequently reported in pediatric individuals. Including our case, 7 of 14 reported cases occurred in dogs <2-y-old, and all but 1 had polyostotic disease. Long bones, ribs, and vertebrae were affected most often, and the distal metaphyseal region was targeted in long bones. Visceral and nodal tissue infiltration was common, and all tumors had a diffuse architecture. Most dogs with polyostotic lymphoma were euthanized at the time of diagnosis, and survival was <6 wk in dogs that were treated with chemotherapy or surgery.

## Case report

An 8-mo-old, 32.5-kg, intact male Mastiff-cross dog was presented to a primary care veterinarian with a 1-mo history of lethargy, progressive hyporexia, and waxing and waning soft stools. During the week prior to presentation, the dog had also developed shifting lameness. Physical examination found mild muscle wasting and swelling around the carpal joints. Pain was elicited by palpation of the carpal joints. Lymph nodes were not enlarged, and there were no abnormalities on abdominal palpation or cardiac auscultation. The dog was noted to be mildly pyretic (39.2°C). A CBC showed mild anemia, lymphocytosis, and thrombocytopenia (Table 1). Serologic assays for dirofilariosis, anaplasmosis, ehrlichiosis, and borreliosis were negative. Serum biochemistry results were unremarkable.

**Table 1.** Hematologic results from a dog with polyostotic lymphoma.

	Day 1*	Day 9*	Day 11†	RI†
Hematocrit, L/L	**0.32**	**0.25**	**0.33**	0.39–0.56
Hemoglobin, g/L	**111**	**88**	**99**	133–197
MCV, fL	68	**63**	**76**	66–75
MCHC, g/L	347	355	101	321–360
RDW, %	**14.7**	**16.4**	**14.4**	11–14
WBC, ×10^9^/L	14.0	9.9	8.7	4.9–15.4
Neutrophils, ×10^9^/L	4.1	3.3	3.6	2.9–10.6
Lymphocytes, ×10^9^/L	**9.7**	**5.6**	4.5	0.8–5.1
Monocytes, ×10^9^/L	0	0.5	0.2	0–1.1
Platelets, ×10^9^/L	**116**	**55**	**56**	117–418
MPV, fL	NR	**20**	**26**	7–14
Reticulocytes, ×10^9^/L	75	52	**102**	0–80
Rubricytes, ×10^9^/L	NR	**1.8**	**4.1**	0

NR = not reported. Results outside their RI are in bold.

* ProCyte analyzer; Idexx Reference Laboratory.

† Advia 2120 analyzer; Animal Health Laboratory, University of Guelph.

**Figure 1.**
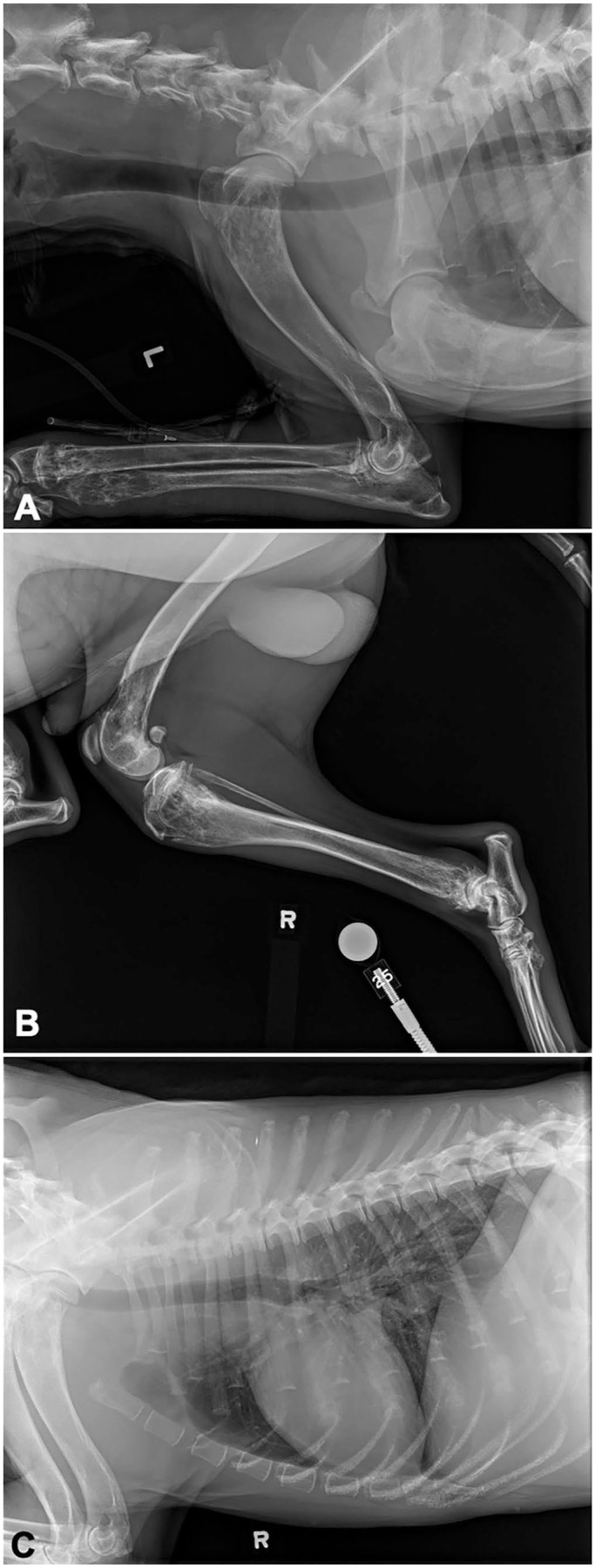
Radiographs from a dog with polyostotic B-cell lymphoma. **A.** Lytic-proliferative bone lesions of distal radius and proximal ulnas. **B.** Similar lesions in proximal and distal tibia and distal femur. **C.** Similar lesions in ribs near costochondral junctions and in dorsal spinous processes.

A 1-wk course of meloxicam (0.1 mg/kg q24h PO) was prescribed, with minimal improvement in clinical signs. Nine days later, the dog was re-presented with worsening lameness and lethargy, and continuing pyrexia (39.9°C). Palpation of the pelvic limbs elicited pain. Radiographs of all limbs showed polyostotic aggressive osseous lesions centered on the metaphyses of the appendicular skeleton, as well as in the thoracic spinous processes, cervical vertebrae, and costochondral junctions (Fig. 1). The differential diagnoses for the radiographic abnormalities were neoplasia, osteomyelitis, and less likely, fibrous dysplasia. CBC was repeated and showed worsening anemia, thrombocytopenia, and rubricytosis, and persistent mild lymphocytosis.

**Figure 2.**
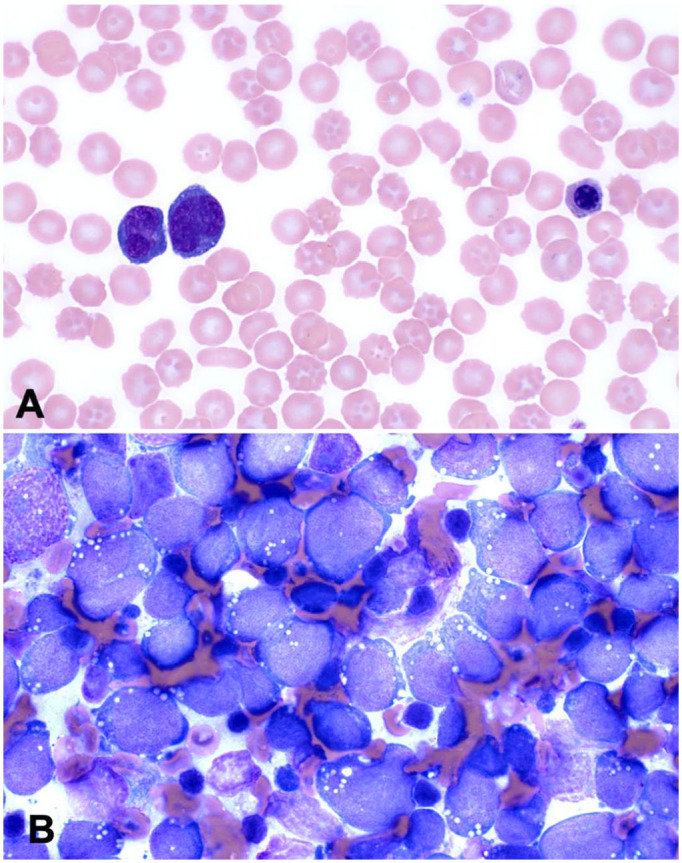
Polyostotic B-cell lymphoma in a dog. **A.** Blood film with large, atypical lymphocytes and a rubricyte. **B.** Cytology preparation of an aspirate from the proximal tibia. The neoplastic lymphocytes are large with intensely basophilic cytoplasm, frequent cytoplasmic vacuoles, and round-to-irregular nuclei.

The dog was referred to the Ontario Veterinary College Teaching Hospital (Guelph, Ontario, Canada) for further evaluation. On presentation, the dog was clinically stable with normal vital signs but pyrexic (39.7°C). The carpi, distal tibiae, and distal humeri bilaterally were swollen and painful. The popliteal and prescapular lymph nodes were enlarged. Thoracic auscultation and abdominal palpation were unremarkable. With point-of-care ultrasonography of the thorax and abdomen, neither free fluid nor lung surface or hollow visceral changes were noted. Venous blood gas analysis showed mild ionized hypercalcemia (1.46 mmol/L; RI^1^: 0.91–1.45 mmol/L). Findings on a repeat CBC were similar as previous (Table 1). Blood film review indicated more marked rubricytosis, and ~50% of lymphocytes were intermediate to large and had atypical morphology consisting of convoluted nuclear shapes and marked cytoplasmic basophilia (Fig. 2A). Serum biochemical analysis found hypoglobulinemia (18 g/L; RI: 21–42 g/L) and a mild increase in creatine kinase activity. A urine sample collected by cystocentesis was concentrated (specific gravity 1.043) and had hematuria and proteinuria. Radiographs of the thorax and forelimbs were repeated with similar findings as before. Detailed abdominal ultrasonography found marked splenomegaly and mildly enlarged medial iliac lymph nodes. The right and left tibia, popliteal lymph nodes, and the left prescapular lymph node were aspirated for cytologic evaluation. Tibiae were chosen because a lytic area was visible by ultrasonography with minimal overlying soft tissue. Preparations from all sites were cytologically similar and consisted of intermediate-to-large lymphocytes with finely granular chromatin, single-to-multiple nucleoli, fine cytoplasmic vacuoles, and frequent mitotic figures (Fig. 2B). The interpretation was lymphoma.

Because there were atypical cells in circulation, a blood sample was analyzed by flow cytometry with a panel of 15 antibodies reactive with canine leukocyte antigens (Suppl. Table 1). Blood lymphocytes were of variable size, uniformly positive for CD45, and moderately positive for CD18, CD21, and MHC class II (Suppl. Figs. 1–3). Approximately 10% of lymphocytes were CD3-, CD4-, CD5-, or CD8-positive T cells. These findings were most consistent with B-cell leukemia, which, in light of the concurrent cytologic diagnosis of lymphoma, was considered to reflect disseminated B-cell lymphoma.

Based on the severity and extent of pain, and the high likelihood of a poor response to therapy, combination chemotherapy was declined, and palliation with corticosteroids was elected. The patient was discharged with oral prednisone (1.5 mg/kg q24h) and gabapentin (9.2 mg/kg q8h) for analgesia. Due to the lack of improvement in pain control, and progressive immobility and hyporexia, the patient was euthanized 7 d after discharge. The body was frozen (–20°C) and submitted for postmortem examination 2 d later.

**Figure 3.**
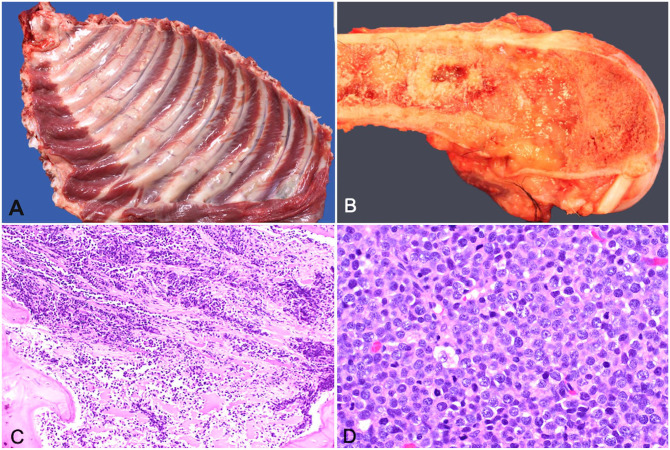
Polyostotic B-cell lymphoma in a dog. **A.** Gross appearance of bony proliferations at the costochondral junctions of ribs. **B.** Medullary infiltrates with lysis of cortical bone of the distal femur. **C.** Section from the proximal tibia with bone lysis and replacement by neoplastic lymphocytes. H&E. **D.** The popliteal lymph node is replaced by sheets of large lymphocytes with interspersed single small lymphocytes and macrophages, and occasional plasma cells. The large lymphocytes have round nuclei, peripheralized chromatin, and single or occasionally multiple nucleoli. H&E.

On postmortem examination, multiple, pale, tan-to-white, gelatinous, round-to-ovoid masses replaced and effaced bone at numerous sites, including the ribs at the level of the costochondral junction (Fig. 3A), the right distal femur (Fig. 3B), the proximal and distal aspects of both the left and right tibia, and multiple vertebral spinous processes. The spleen and liver were diffusely enlarged.

Histologically, densely packed sheets of pleiomorphic round cells occupied and effaced cortical and trabecular bone of the right femur and scapula, both tibiae, multiple ribs, and cervical and thoracic spinous processes (Fig. 3C, 3D). Similar cells extensively infiltrated the medullary space of the bone, the right prescapular, right popliteal, left popliteal, left submandibular, colonic, right submandibular, and mesenteric lymph nodes, parenchyma of the spleen, dissected between sinusoids and surrounded the central vein and periportal regions of the liver, and were within the lumen of vessels in the lungs. The neoplastic cells had variably distinct cell borders, a moderate amount of basophilic cytoplasm, and round nuclei with a diameter 2–3 times the size of an erythrocyte. The nuclei had clumped chromatin and 1–3 prominent magenta nucleoli. There was up to 5-fold anisocytosis and anisokaryosis, and 61 mitotic figures per 2.37 mm^2^. One section of marrow from the right radius had a moderate amount of extracellular amorphous eosinophilic material, which was considered to be necrosis rather than amyloid based on negative Congo red staining.

**Figure 4.**
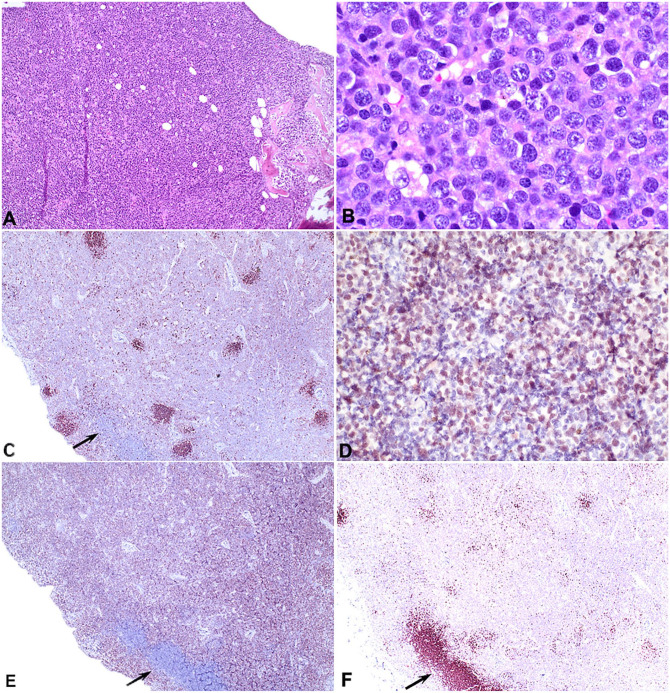
Polyostotic B-cell lymphoma in a dog. **A.** Diffuse sheets of lymphocytes associated with bone lysis. **B.** On higher magnification, the lymphocytes have a high nuclear:cytoplasmic ratio, open chromatin, and a single prominent nucleolus or several less distinct nucleoli. **C.** Diffuse nuclear immunoreactivity among the sheets of lymphocytes, remnant follicles that are intensely immunoreactive, and a cluster of immunonegative lymphocytes (arrow). PAX5 immunohistochemistry (IHC). **D.** At higher magnification, lymphocytes have nuclear immunoreactivity. PAX5 IHC. **E.** Diffuse cytoplasmic immunoreactivity in the neoplastic lymphocytes and a cluster of immunonegative lymphocytes (arrow). CD20 IHC. **F.** Cluster of lymphocytes that were PAX5 and CD20 negative (arrow) and a few single cells in remnant follicles. IHC for CD3.

Immunohistochemical assays for CD3, CD20, PAX5, and MUM1 were applied to sections from a mesenteric lymph node and the right tibia (Table 2). The neoplastic round cells were positive for PAX5 and CD20, and negative for CD3 and MUM1 (Fig. 4). Control sections yielded appropriate results. These immunohistochemical findings indicated B-cell lymphoma with the histomorphology of diffuse large B-cell lymphoma (DLBCL). The extensive bone effacement, multicentric distribution, and relatively modest neoplastic lymphocytosis and marrow infiltration were interpreted as most consistent with primary bone lymphoma (PBL) and secondary soft tissue infiltration.

**Table 2.** Reagents and parameters for immunohistochemical assays.

Marker	Antibody type	Source	Clone or ID	Antigen retrieval*	Dilution
CD3	Polyclonal, rabbit	Dako	A0452	pH 6	1:100
CD20	Polyclonal, rabbit	LabVision	RB-9013	pH 6	1:200
PAX5	Monoclonal, mouse	Biocare Medical	24	pH 9	1:50
MUM1	Monoclonal, mouse	Dako	MUM1p	pH 8	1:50

Bound antibodies were detected with Nova Red chromogen (Dako).

* Heat-induced.

## Discussion and literature review

Lymphoma involving bone is rare in humans and animals. PBL was initially defined as single or multiple bone lesions without lymph node or visceral involvement.^4^ Because ruling out regional lymph node involvement can be challenging, the definition was subsequently amended to encompass 3 different stages of PBL: solitary bony lesion, solitary bony lesion with regional lymph node involvement, and multifocal disease.^26^ None of the current classification systems for human lymphoid neoplasms include PBL as a distinct entity, although unique phenotypic and genetic features of PBL have been identified.^8^

PBL refers to neoplastic lymphocytes in bone itself, and should be differentiated from intramedullary lymphoid neoplasia, which can reflect marrow infiltration by stage V peripheral lymphoma, lymphocytic leukemia arising in hematopoietic tissue, and primary bone marrow lymphoma.^21^ Stage V peripheral lymphoma arises in lymph nodes or extranodal sites, and infiltrates marrow as the disease progresses. Lymphocytic leukemia arises in hematopoietic marrow and is associated with neoplastic lymphocytosis. During disease progression, peripheral organs and lymph nodes are gradually infiltrated.

Dogs with primary bone marrow lymphoma have neither organ, bone, or lymph node enlargement nor overt leukemia at the time of diagnosis, are often hypercalcemic, and the neoplasm is composed of CD4+ T cells.^18,20^ Our case had extensive polyostotic disease prior to detection of lymph node enlargement. Disease progression was rapid, and at the time of euthanasia, it was not possible to ascertain whether neoplastic bone lesions had preceded organ involvement. However, bone infiltration and lysis are rarely features of multicentric nodal or extranodal lymphoma in dogs. Therefore, our case of lymphoma was considered to be distinct, and most consistent with PBL that had disseminated and progressed to secondary nodal and organ involvement.

In humans, PBL comprises 3–7% of primary bone cancers and <1% of all lymphomas.^4,9^ PBL is more common in children than in adults, and less common than other pediatric primary bone tumors such as osteosarcoma and Ewing sarcoma. Skeletal pain was the most common symptom in humans of all ages; bone lysis was detected on radiography in 59% of cases, a single site was more commonly affected than multiple sites, and ~30% of cases required >1 biopsy for diagnosis.^4,15^ Histologically, 75–85% of human pediatric and adult cases of PBL are classified as DLBCL based on World Health Organization (WHO) criteria. Under the large umbrella of DLBCL, PBL (also called primary non-Hodgkin lymphoma of bone and primary bone diffuse large B-cell lymphoma) is recognized as a clinically but not histomorphologically distinct entity. The less-common WHO subtypes among PBL are anaplastic large-cell lymphoma, low-grade follicular lymphoma, and others.^15^

We identified 13 cases of lymphoma affecting bone in dogs published since ~1990 (Table 3).^1,5–7,10–12,16,22–25^ Six other cases were reported in abstract format.^17^ Of the published cases, one was considered to have multicentric T-cell lymphoma with paraneoplastic diffuse idiopathic spinal hyperostosis rather than PBL.^6^ Two dogs had extensive nodal and visceral involvement by lymphoma and lesions in several ribs, which was interpreted as multicentric lymphoma with secondary bone involvement.^5,16^ One case with intramedullary lymphoma and lysis in a vertebra and femur without bony proliferation or evidence of soft tissue involvement was more alike to primary bone marrow lymphoma than PBL.^11,17,19^ Seven of the remaining 9 cases occurred in dogs <2-y-old with radiographic lesions similar to our case. Of these, 1 dog had a monostotic lesion and was treated with radiation that resulted in survival of >2.9 y, and 6 dogs had polyostotic disease and soft tissue involvement.^1,7,12,22,23^ Of these 6 dogs, 1 had a large thymoma and lesions in both femurs and humeri but not in viscera.^1^ Four of 7 dogs with likely PBL were 4–10-y-old.^5,10,16,24^ Although these case numbers are small, they nevertheless suggest that PBL may be more common in juvenile than adult dogs, as is the case in humans.

**Table 3.** Historical cases of canine bone lymphoma.

Age, y	Sex	Breed	Bone site	Non-bone site	Type	Treatment	Outcome
1.5	CM	Boxer	Ulna	None	Diffuse	Radiation	Alive at 2.9 y^7^
0.9	SF	Basset	Multiple	Spleen, kidney, liver	Diffuse	None	Euthanasia at diagnosis^22^
0.8	F	German Shepherd	Multiple	Spleen, liver, thymus	Diffuse, T/B inconclusive	None	Euthanasia, 4 wk^12^
0.5	M	Golden Retriever	Multiple	None	Diffuse	None	Euthanasia at diagnosis^23^
1.7	F	Golden Retriever	Vertebrae	LN, spleen, liver, BM	Diffuse, CD3^–^/4^+^/79^+^	None	Euthanasia at diagnosis^25^
0.3	F	Golden Retriever	Multiple	LN, liver, kidney	Diffuse	None	Euthanasia, 4 d^20^
0.5	F	German Shepherd	Femurs, humeri	Thymus	Diffuse, T, CD4^+^/8^+^	None	Euthanasia at diagnosis^1^
4.0	F	Whippet	Femur, humerus	LN, heart, liver, spleen, kidney	Diffuse	CHOP	Death, 10 d^16^
7.0	SF	Siberian Husky	Multiple	NR	Diffuse	Radiation, CHOP	Euthanasia, 2 wk^24^
9.0	SF	Scottish Terrier	Vertebrae	LN, liver	NR	Prednisone, L-asparaginase	NR^10^
9.0	CM	Golden Retriever	Ribs	LN, spleen, liver, kidney, intestine	Diffuse	None	Euthanasia at diagnosis^5^
10.0	M	Mixed breed	Vertebral and femur marrow	None	Diffuse, T	Surgery	Euthanasia, 6 wk^11^
4.0	SF	Bull Mastiff X	Vertebral hyperostosis	LN, thymus, liver, spleen, stomach	Diffuse, T, CD3^+^/5^–^	None	Euthanasia at diagnosis^6^

BM = bone marrow; CM = castrated male; F = female; M = male; LN = lymph node; NR = not reported; SF = spayed female.

Pain and bone lysis were the most common clinical features in canine PBL. As in humans, the diagnosis of PBL in dogs was often delayed due to initial consideration of more common conditions associated with bone pain and lysis, such as osteomyelitis or osteosarcoma. In humans, monostotic disease was more common than polyostotic disease. However, in dogs, only 1 of 14 reported cases had monostotic disease, and polyostotic disease, as in our case, appears to be more common. It is uncertain whether this reflects diagnosis later in the course of disease in dogs relative to humans or species differences in tumor biology. Predilected sites for PBL in dogs are difficult to determine, but long bones (diaphyseal region) were reported as more frequently affected than flat bones. The meta-diaphyseal region of long bones is frequently affected in humans,^8^ and was also affected in our case.

Descriptions of histomorphologic features of PBL in dogs are limited. When reported, bone was replaced with diffuse sheets of small-to-large lymphocytes with frequent periosteal infiltration, bone remodeling, pathologic fractures, variable marrow effacement, and extra-skeletal involvement.^7,12,25^ The number of mitotic figures was interpreted as high in most cases, although enumeration per area was not performed. Immunophenotyping by flow cytometry or immunohistochemistry (IHC) was suggestive of T-cell lymphoma in 2 cases each, and as DLBCL based on morphology in another case.^7,12,25^ The immunophenotyping results were often ambiguous and did not assess lesions in bone.^25^ One case was interpreted as DLBCL based on morphology without IHC.^7^ Using flow cytometry, another case was reported as CD4 and CD79a positive; these are usually mutually exclusive markers of T and B cells, respectively. The 2 cases considered to be unequivocally T cell were the dog with thymoma and bone lesions, and the case most similar to marrow lymphoma.^1,11^ IHC characterization of neoplastic lymphocytes should include T- and B-cell markers and ideally more than one of the B-cell markers PAX5, CD20, and CD79A. Detection of intracellular antigens such as CD79A by flow cytometry is prone to nonspecific results. Results of IHC in our case indicated B-cell type based on the location and proportion of PAX5- and CD20-positive cells on IHC. The neoplastic cells were faintly positive for PAX5 compared to intensely positive remnant follicles in the same section (Fig. 4C). CD20 IHC results were more pronounced and uniform than PAX5 IHC.

Hematologic changes in dogs with PBL are rarely described. The dog in our case was anemic and developed thrombocytopenia over the course of 11 d; hematopoietic marrow was replaced by neoplastic lymphocytes in areas adjacent to proliferative bone lesions and was infiltrated with neoplastic lymphocytes in areas with less prominent bone proliferation. Lymphocytosis was mild but included morphologically abnormal cells. Involvement of bone marrow by PBL was reported in 5 of the historical cases, and may not have been evaluated in the remaining cases. In our case, atypical blood lymphocytes were interrogated with a panel of 15 concurrent antibodies to investigate the immunophenotype of the presumed neoplastic circulating cells. The lymphocytes were moderately positive for CD18 and CD21, heterogeneously positive for CD45, and uniformly positive for MHC II (Suppl. Figs. 1–3). Other markers were detected on <2% of small lymphocytes. These findings were consistent with circulating neoplastic B cells, although more uniform and pronounced positivity for CD21 is expected in health and in B-cell chronic lymphocytic leukemia.^3,14^ Analysis of a sample aspirated from a lytic lesion may have allowed for better comparison with IHC.

Treatment of PBL in dogs has been attempted with single and combination chemotherapy, radiation, and surgery. Without uniform diagnostic criteria and therapy, prognostic features for PBL cannot be derived; however, none of the dogs with polyostotic disease lived >6 wk even though chemotherapy was administered in 3 cases.^10,16,24^ Survival of ~3 y was reported for the dog with monostotic ulnar lymphoma.^7^ In humans, PBL of DLBCL type is most often treated with radiation ± immunotherapy or chemotherapy if monostotic, and with combination chemotherapy ± immunotherapy if polyostotic.^15^ Five-year survival of 65–70% has been reported from larger studies of humans, although most cases had monostotic disease, and treatment was also non-uniform. Our case had an extensive bone and extra-skeletal tumor burden, cytopenia, and severe pain, and was considered to have advanced and widely disseminated disease that was unlikely to respond favorably to combination chemotherapy.

Polyostotic bone lymphoma has been reported in a 0.5-y-old cat and an 8.5-y-old ferret.^2,13^ The cat had CD20-positive B-cell lymphoma in multiple long bones, infiltrative disease in lymph nodes, marrow, and viscera, as well as hypercalcemia, clonal gammopathy, and clonal B-cell antigen receptor rearrangement. The ferret had grossly proliferative lesions in both humeri, a mandible, and a femur, and infiltrative disease in lymph nodes, viscera, and skeletal muscle. The neoplastic cells were CD3 positive and CD79a negative, and marrow was extensively effaced; the bone lesions were interpreted as likely secondary to primary marrow lymphoma. These single reports of bone lymphoma convey that the disease is likely rare and heterogeneous across species.

## Supplemental Material

sj-pdf-1-vdi-10.1177_10406387251329020 – Supplemental material for Canine polyostotic B-cell lymphoma: a case with clinical, immunohistochemical, and flow cytometric characterization, and review of the literature Supplemental material, sj-pdf-1-vdi-10.1177_10406387251329020 for Canine polyostotic B-cell lymphoma: a case with clinical, immunohistochemical, and flow cytometric characterization, and review of the literature by Matthew Kornya, Connor Bryant, Brandon Lillie, Sebastien Sanz, Kristiina Ruotsalo and Dorothee Bienzle in Journal of Veterinary Diagnostic Investigation
