# Supplementary material for: Canine polyostotic B-cell lymphoma: a case with clinical, immunohistochemical, and flow cytometric characterization, and review of the literature
Source: J Vet Diagn Invest. 2025 Mar 21;37(4):697–704. doi: 10.1177/10406387251329020 (PMC11948266; doi:10.1177/10406387251329020)
Supplement: sj-pdf-1-vdi-10.1177_10406387251329020 – Supplemental material for Canine polyostotic B-cell lymphoma: a case with clinical, immunohistochemical, and flow cytometric characterization, and review of the literature [file sj-pdf-1-vdi-10.1177_10406387251329020.pdf]

JVDI: Supplemental material

Kornya M, et al. Canine polyostotic B-cell lymphoma: a case with clinical, immunohistochemical, and flow cytometric characterization, and review of the literature

Supplemental Table 1. Flow cytometry antibody panel. All antibodies were individually titrated and then tested in combination. Fluorescence was assessed in a spectral flow cytometer (Northern Lights; Cytek) with a 16-color concurrent analysis.

Antigen	Fluorochrome	Clone	Source
NA	Zombie Violet*	NA	BioLegend
CD3	Alexa Fluor 700	CA17.2A12	Bio-Rad
CD4	StarBright Violet 570	YKIX302.9	Bio-Rad
CD5	PerCP-eFluor 710	YKIX322.3	ThermoFisher
CD8a	Super Bright 702	YCATE55.9	ThermoFisher
CD11c	Allophycocyanin/Cyanine 7	N418	BioLegend
CD11b	Phycoerythrin/Cyanine	M1/70	Southern Biotech
CD14	PE-Vio 615	REA599	Miltenyi
CD18	StarBright Blue 700	YFC118.3	Bio-Rad M
CD18	Alexa Fluor 700	CA1.4E9	Bio-Rad
CD21	Brilliant Violet 650	B-ly4	BD Biosciences
CD25	Super Bright 600	P4A10	ThermoFisher
CD34	Phycoerythrin	1H6	Abcam
CD45	Alexa Fluor 700	YKIX716.13	Bio-Rad
CD80	Brilliant Violet 421	16-10A1	BioLegend
MHC II	StarBright Violet 515	YKIX334.2	Bio-Rad

NA = not applicable.

* Viability fluorochrome.

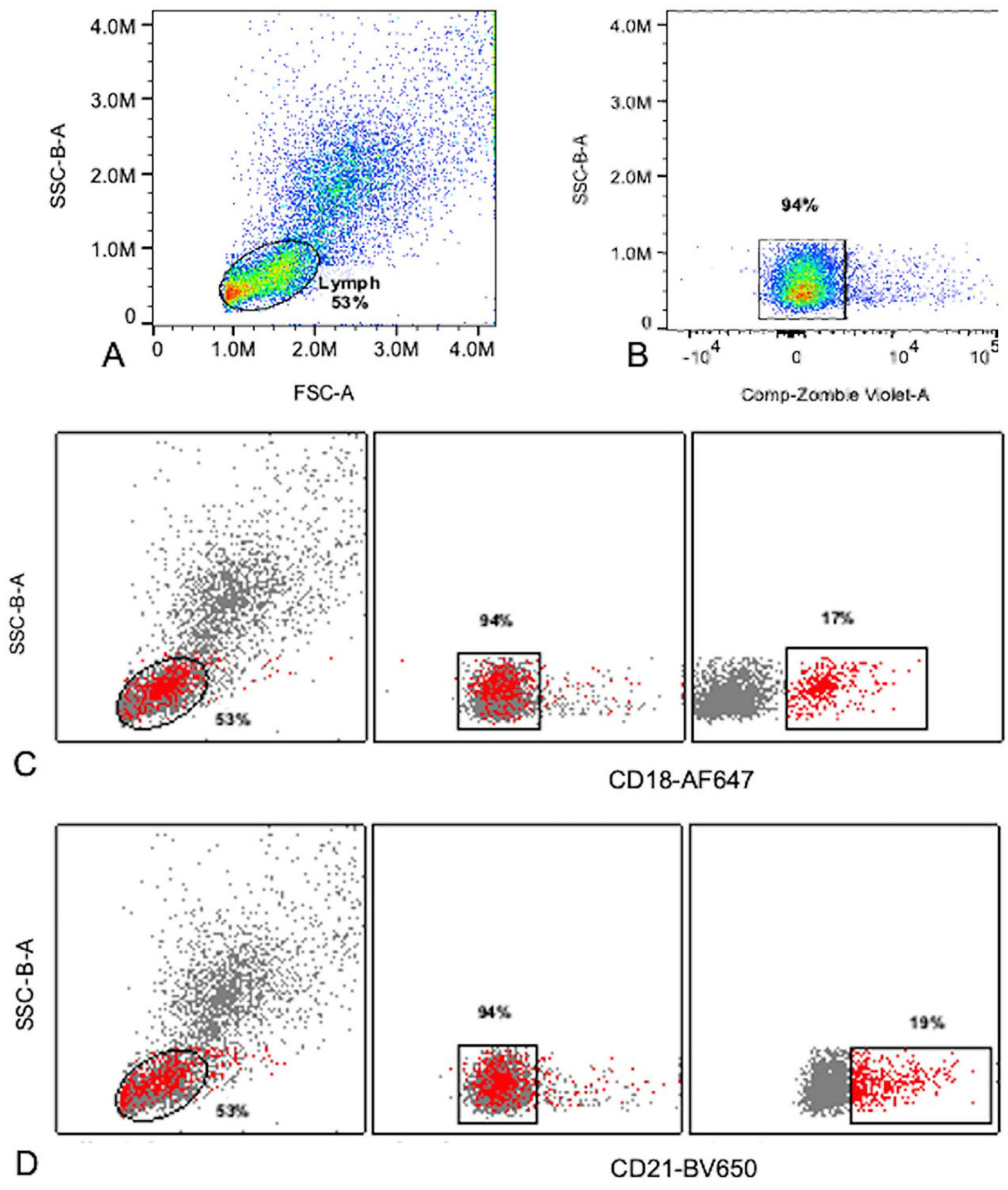

Supplemental Figure 1. Polyostotic B-cell lymphoma in a dog. **A.** Forward (FSC) and side (SSC) light scatter of blood leukocytes; ~53% fall into the lymphocyte gate. **B.** 94% of gated cells are viable (Zombie Violet negative). **C.** Backgating indicates that 17% of viable cells in the lymphocyte gate (red) are CD18+ (x-axis; clone CA1.4E9). **D.** Approximately 19% of cells are CD21+ (x-axis). CD21 positivity ranges from dim to bright.

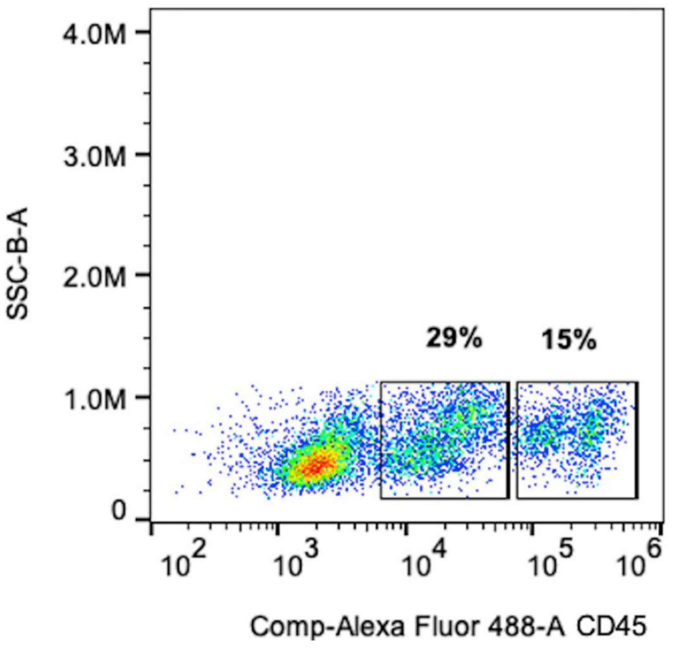

Supplemental Figure 2. Polyostotic B-cell lymphoma in a dog. CD45 fluorescence shows negative, dim positive (29%), and bright positive (15%) viable cells in the lymphocyte gate.

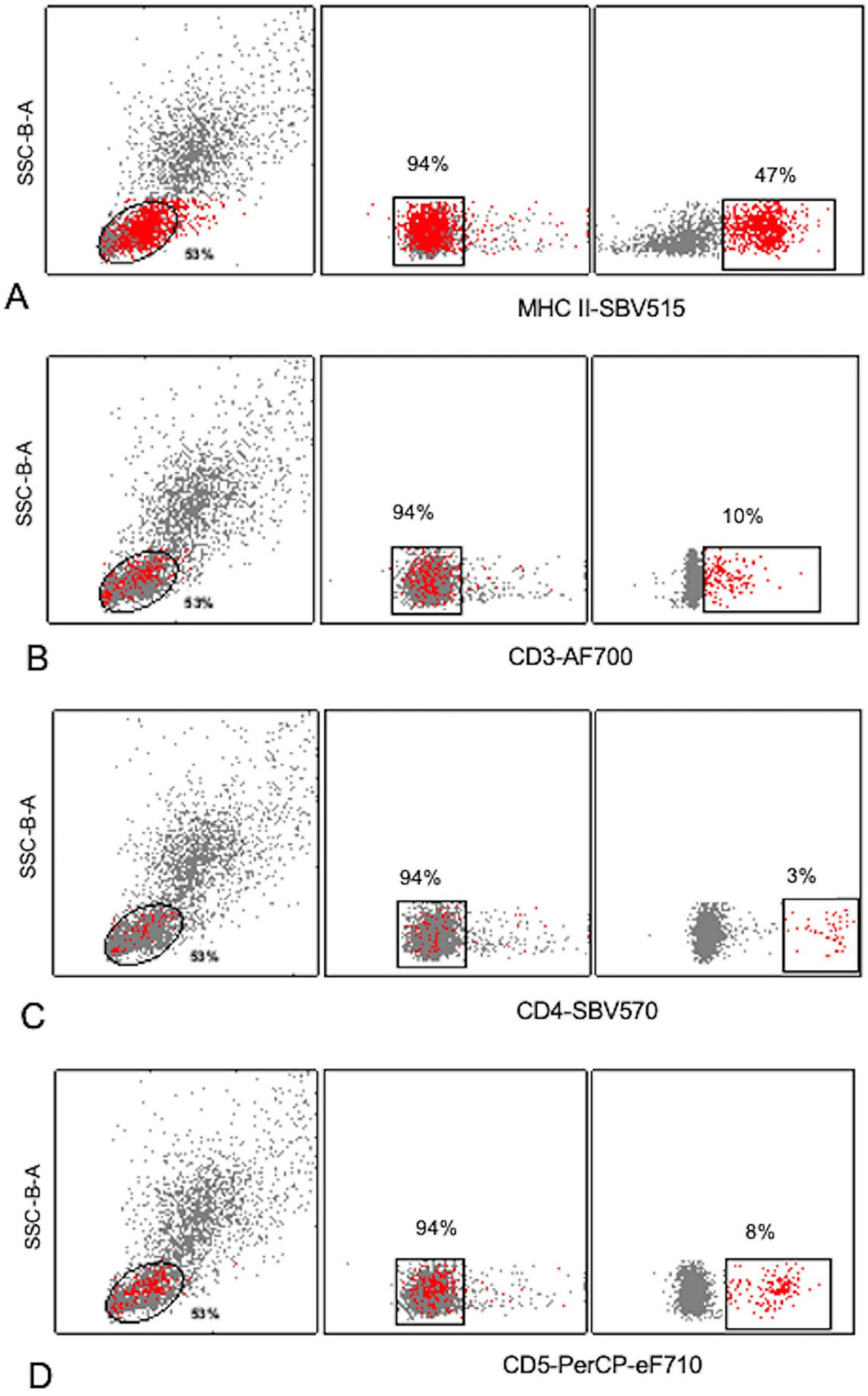

Supplemental Figure 3. Polyostotic B-cell lymphoma in a dog. Of cells in the lymphocyte gate: **A.** 47% are MHC II+; **B.** 10% are CD3+; **C.** 3% are CD4+; **D.** 8% are CD5+.
